# The Heart of the British People

**Published:** 1914-08-29

**Authors:** 


### The Hospital

The Workers' Newspaper of Administrative Medicine and Institutional Life, Administration, National Insurance and Health.

No. 1471, Vol. LVI. SATURDAY, AUGUST 29, 1914. PRICE ONE PENNY.

### THE HEART OF THE BRITISH PEOPLE.

In the appeal to the nation issued by the Prince of Wales on the 6th instant he referred to "the present time of deep anxiety" and truly declared: "At such a moment we all stand by one another. It is to the heart of the British people I confidently appeal." It has been essential for prudent people to weigh well and deeply the problem of the employees called to the war, and the words of His Royal Highness. The interests of the employer, the employee, and the wives or widows and children of the latter are closely bound up together. Assuming that the Prince's words have found an echo in every British heart, we wish to elucidate and remove various difficulties which have presented themselves.

The employers'—that is the institutional—case has caused much discussion as to the best course to follow in the interests of everybody. It would be worthy of the hour and a united nation to secure that all hospital authorities should act uniformly in this matter of the pay and remuneration of their employees, who may be with the Colours. In arriving at a wise solution it is essential that all the interests involved should be clearly understood. The first step for each committee to take would seem to be to ascertain, either from the War Office or the Admiralty or through the Soldiers' and Sailors' Families' Association, exactly what pay is given by each of the Services to all branches of each. Next it is important to understand clearly whether the employee is married or single. A large and increasing number of individual employers are continuing full pay to the absentees with the Colours. This course may directly aid Lord Kitchener by encouraging eligible men to serve, and may be commended on this ground. But justice in the case of the hospitals can alone be adequately met in the special circumstances, by each committee ascertaining, in the case of married men having wives and children dependent on them, how best to arrange the emoluments given to the absentee, in addition to his military pay, as to prevent his family from suffering avoidable distress and need. Some committees have adopted the principle of giving half-pay to all men with the Colours, which might prove a handsome arrangement for single men, but possibly quite inadequate to meet the case of some, at any rate, of the men who are married. Another plan has been to make the pay received by the sailor or soldier up to a sum equal to the weekly emoluments paid to him as an employee in civil life.

In coming to a decision, each committee should try to realise the full seriousness of the crisis which the nation and the Empire have to face and weather. Every committee that does this will not be content to adopt some relatively mean and inadequate plan on economic grounds. Our hospitals have made great sacrifices and are doing great things for the country by the spirit with which they have set themselves to meet the hospital requirements for the sick and wounded imposed by a state of war. But in regard to their employees hospital committees will we hope, as the directing minds of our Houses of Recovery, determine to respond to the Prince of Wales's national appeal by making the emoluments and circumstances of every one of their employees absent at the war, always adequate to prevent any possibility of their wives and children requiring any aid from the Prince of Wales's National Fund. We hope, therefore, that every committee may respond in the most practical way possible to the Prince of Wales's appeal by adopting the policy here indicated.

Again, every hospital officer, every nurse, and every employee throughout the whole of the institutions of Great Britain and Ireland, who remains at the post of duty within the hospitals and institutions, will naturally desire each to do his or her part to help allay the anxiety of our sailors and soldiers when on service, and to prevent distress to the wives and families which they leave behind them. It is not easy to determine exactly how each worker can best render practical help to the Prince of Wales's National Fund. We have given a good deal of consideration to the point, and have come to the conclusion that, providing the chief officers will assemble all the employees in each hospital, convalescent institution, nursing home, and sick-house throughout the country, and confer with them, they can readily learn the sum which each will contribute monthly to the Prince of Wales's Fund. The result should prove of the first importance to the National Fund. Everybody everywhere, and especially the women, is clamouring for something to do in order that each may in some way help the sick and wounded. Hence the spontaneous exhibition of an organised effort to unite everybody employed in the service of the sick and injured who may be working in institutions throughout the land, would stimulate public efforts for the National Fund of which the Prince of Wales is the treasurer.

To make such spontaneous action attain its maximum efficiency, it is essential that some officer, preferably one for the female and another for the male staff, should be appointed treasurer for each institution; next that the actual monthly contribution agreed to be subscribed, with the names of each hospital, institution, and individual subscriber, should be recorded on one list, and that that list should be published in its entirety. If our idea proves generally acceptable to the workers throughout British institutions, we will gladly undertake to keep a Great Book of Remembrance in which all the names and contributions from each institution can be entered, and to publish particulars as they are sent in to the Editor. The secretary or some representative of each committee should be appointed to receive these spontaneous subscriptions by the staff, and to send them periodically to the Prince of Wales, at Buckingham Palace, that they may be duly credited to the National Fund. By some such simple organisation every institution throughout the country that already has the privilege of ministering to the sick and injured could directly relieve the National Fund from considerable expenditure. It could add materially to the total contribution raised for this Fund by the British people under the leadership of the Heir Apparent to the throne. The Prince's belief that we all stand by one another, and that the heart of the British people is sound, might thus be strikingly demonstrated. Such spontaneous and united action by all British workers for the sick and injured must prove indeed memorable in the history of the English people.

